# Impact of Exercise and Detraining during Childhood on Brown Adipose Tissue Whitening in Obesity

**DOI:** 10.3390/metabo11100677

**Published:** 2021-10-01

**Authors:** Kaho Takaishi, Takaya Oshima, Hikari Eto, Misuzu Nishihira, Son Tien Nguyen, Ryosuke Ochi, Naoto Fujita, Susumu Urakawa

**Affiliations:** Department of Musculoskeletal Functional Research and Regeneration, Graduate School of Biomedical and Health Sciences, Hiroshima University, 1-2-3 Kasumi, Minami-ku, Hiroshima 734-8553, Japan; kaho-takaishi@hiroshima-u.ac.jp (K.T.); takaya-oshima@hiroshima-u.ac.jp (T.O.); b180622@hiroshima-u.ac.jp (H.E.); m-nishihira@hiroshima-u.ac.jp (M.N.); d193461@hiroshima-u.ac.jp (S.T.N.); ryosuke-ochi@hiroshima-u.ac.jp (R.O.); urakawas@hiroshima-u.ac.jp (S.U.)

**Keywords:** obesity, brown adipose tissue, exercise, childhood

## Abstract

This study aimed to investigate the influence of childhood exercise and detraining on brown adipose tissue (BAT) whitening in obesity. Four-week-old male Long-Evans Tokushima Otsuka (LETO) rats (*n* = 9) and Otsuka Long-Evans Tokushima Fatty (OLETF) rats (*n* = 24) were used as non-obese and obese animals, respectively. OLETF rats were divided into non-exercise sedentary (*n* = 9) and exercise groups. OLETF rats in the exercise group were further divided into subgroups according to the exercise period—exercise from 10- to 12-weeks-old (*n* = 6); and exercise from 4- to 6-weeks-old, and detraining from 6- to 12-weeks-old (*n* = 9). At 12-weeks-old, immediately after exercise period, BAT whitening in OLETF rats was inhibited by exercise despite the fact that hypertrophy was not caused in the plantaris muscle. However, the effectiveness was attenuated during the detraining period. Histological BAT whitening and downregulation of uncoupling protein-1 (UCP-1) were found in non-exercise sedentary OLETF rats at 12-weeks-old. The downregulation was not inhibited, even though exercise histologically inhibited BAT whitening in OLETF rats. Childhood exercise decreased BAT whitening in obesity. Detraining attenuated the inhibition of BAT whitening. These results suggest that regular exercise is needed to improve BAT whitening and downregulation of UCP-1 in obesity.

## 1. Introduction

Adipose tissues are composed of two major types: white adipose tissue (WAT) and brown adipose tissue (BAT), which have different features. White and brown adipocytes are histologically characterized as containing unilocular and multilocular lipid droplets, respectively [1]. WAT has a function of energy storage in the lipid droplets, while BAT dissipates the lipid droplets to produce heat [2,3]. Brown adipocytes possess smaller lipid droplets and numerous mitochondria, which contribute to a high rate of fatty acid oxidation and subsequent heat production. Heat production in BAT depends on the activity of uncoupling protein-1 (UCP-1) [3,4]. UCP-1 is localized in the inner mitochondrial membrane and acts as an uncoupler of oxidative phosphorylation and adenosine triphosphate (ATP) synthesis, thereby dissipating energy as heat [5,6]. The transformation of the lipid depot from multilocular to unilocular has been reported in brown adipocytes in obesity; the change from BAT to WAT is known as whitening [7,8]. Excess and ectopic fat accumulation, including BAT whitening, are major risk factors for the development of hyperlipidemia, type 2 diabetes, and cardiovascular disease [9]. Previous studies have reported that BAT whitening is associated with downregulation of mitochondrial proteins [10,11]. Downregulation of mitochondrial proteins induced by BAT whitening decreases the expression of UCP-1 [10,11]. Downregulation of UCP-1 impairs energy consumption and heat production, which accelerates the progression of lipid accumulation and obesity. Therefore, it is necessary to focus on BAT whitening to prevent and eliminate obesity.

Exercise is effective in preventing and improving metabolic diseases, such as obesity and type 2 diabetes [5,12]. Exercise shrinks hypertrophic brown adipocytes and suppresses BAT whitening in obesity [13,14]. Furthermore, exercise increases mitochondrial activity and improves the thermogenic capacity of brown adipocytes [15,16]. Although the effects of exercise on BAT whitening have been reported, the optimal period for starting exercise remains unknown.

Childhood obesity is a crucial issue that often leads to chronic metabolic diseases such as hypertension, dyslipidemia, insulin resistance, prediabetes, type 2 diabetes, and fatty liver [17]. The presence of obesity from childhood to adolescence is usually carried on to adulthood [18]. Ward et al. reported that approximately 80% of obese children remain obese in adulthood [19]. Similarly, Singh et al. reported that the risk of overweight children remaining obese in adulthood is at least twice that of normal-weight children [20]. Therefore, interventions for obesity during childhood are recommended to decrease morbidity and mortality in adulthood [21,22].

Regular physical exercise promotes positive adaptations in childhood obesity and acts as an adjuvant for prevention and treatment [23,24]. However, the amount of time spent on regular physical activity—including exercise training, sports, and playing outside—tends to decrease gradually from childhood to adolescence [25,26]. The rapid increase in prevalence of obesity is a major concern around the world, and the prevalence of overweight and obesity are recently rising especially in children and adolescents [27]. Therefore, the influence of exercise and non-exercise, such as detraining and sedentary activity in childhood, need to be clarified.

BAT whitening attenuates energy consumption and accelerates the progression of obesity [9,28,29]. Therefore, inhibition of BAT whitening in childhood is important to decrease the incidence of obesity in adulthood. However, the influence of childhood exercise and detraining on BAT whitening remains unknown. Understanding the influence of exercise and detraining on BAT whitening could provide a valuable resource for obesity and the risk of obesity-related complications. The purpose of this study was to investigate the influence of childhood exercise and detraining on BAT whitening in obesity.

## 2. Results

### 2.1. Changes in Body Weight

Four-weeks-old male Long-Evans Tokushima Otsuka (LETO) rats and age-matched male Otsuka Long-Evans Tokushima Fatty (OLETF) rats were used as non-obese and obese animals. Our previous study [30] showed that the body weight of OLETF rats increased from 4-weeks-old to approximately 30-weeks-old when compared to LETO rats. The body weight was significantly higher in OLETF rats than in LETO rats after 6-weeks-old. At 6-weeks-old, the mean body weight in OLETF rats was 1.2-fold higher than LETO rats. Dyslipidemia and glucose intolerance were observed in OLETF rats from 6- to 8-weeks-old [31], which suggest becoming obese rats in the immature stage.

**Figure 1:**
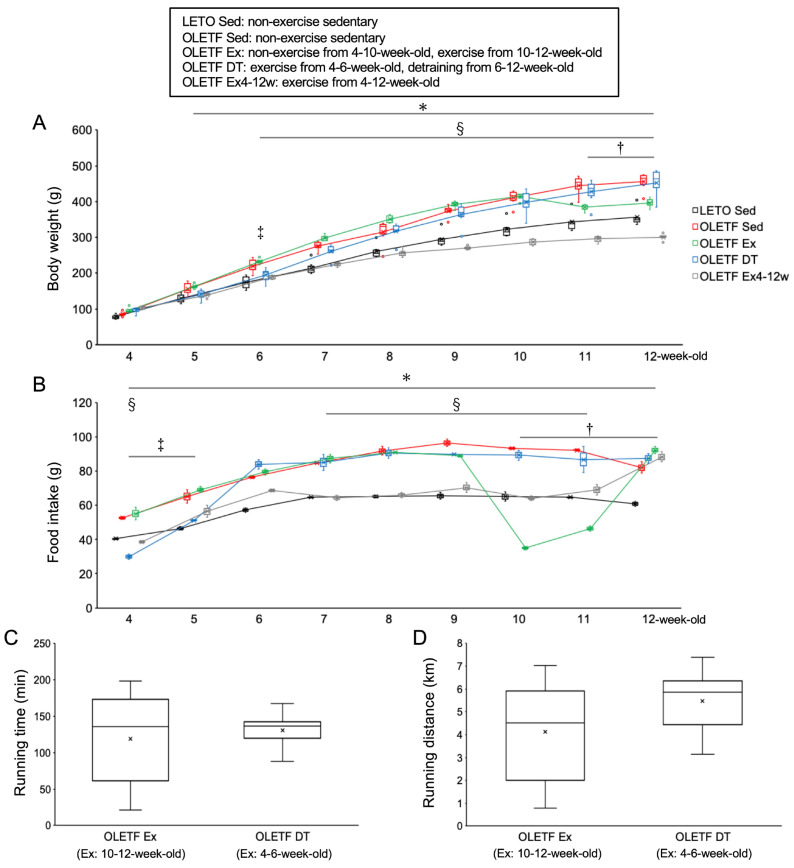
Metabolic characteristics. (**A**) changes in body weight; (**B**) daily food intake (per three rats in a cage); (**C**) daily running time; (**D**) daily running distance. LETO, Long-Evans Tokushima Otsuka rat; OLETF, Otsuka Long-Evans Tokushima Fatty rat. LETO Sed, LETO non-exercise sedentary; OLETF Sed, OLETF non-exercise sedentary; OLETF Ex, OLETF non-exercise from 4- to 10-weeks-old and exercise from 10- to 12-weeks-old; OLETF DT, OLETF exercise from 4- to 6-weeks-old and non-exercise detraining from 6- to 12-weeks-old; OLETF Ex4–12w, OLETF exercise from 4- to 12-weeks-old. Values represent means ± standard deviation. *, †, ‡, and § show significantly different between the OLETF Sed vs. LETO Sed groups, the OLETF Ex vs. OLETF Sed groups, the OLETF DT vs. OLETF Sed groups, and the OLETF Ex4–12w vs. OLETF Sed groups, respectively, *p* < 0.05.

There were no significant differences in body weight between the OLETF non-exercise sedentary (OLETF Sed) and LETO non-exercise sedentary (LETO Sed) groups at 4-weeks-old (*p* = 1.00, Figure 1A). Food intake was significantly higher in the OLETF Sed group than in the LETO Sed group throughout the experimental period (*p* < 0.01, Figure 1B). The body weights in the OLETF Sed group gradually increased throughout the experimental period, and then the value was significantly higher in the OLETF Sed group than in the LETO Sed group after 5 weeks of age (*p* < 0.05). At 12-weeks-old, the mean body weight in the OLETF Sed group was 1.3-fold higher than the LETO Sed group.

There were no significant differences in body weight between the OLETF non-exercise from 4- to 10-weeks-old and exercise from 10- to 12-weeks-old (OLETF Ex) and OLETF Sed groups from 4- to 10-weeks-old (*p* = 1.00). The food intake in the OLETF Ex group was significantly lower at 10- and 11-weeks-old during the exercise period than at 9-weeks-old before the exercise period (*p* < 0.001). Body weight was significantly lower in the OLETF Ex group than in the OLETF Sed group at 11- and 12-weeks-old (*p* < 0.001). Additionally, there were no significant differences in body weight between the OLETF Ex and LETO Sed groups at 12-weeks-old (*p* = 0.41).

There were no significant differences in body weight between the OLETF Ex and OLETF Ex4–12w groups at 4-weeks-old (*p* = 1.00). Body weight was significantly lower in the OLETF Ex4–12w group than in the OLETF Sed group after 6-weeks-old (*p* < 0.05). Additionally, body weight was significantly lower in the OLETF Ex4–12w group than in the OLETF Ex group after 6-weeks-old (*p* < 0.001). At 12-weeks-old, the mean body weight in the OLETF Ex4–12w group was significantly lower than that in the LETO Sed group (*p* < 0.001).

There were no significant differences in body weight between the OLETF exercise from 4- to 6-weeks-old and detraining from 6- to 12-weeks-old (OLETF DT) and OLETF Sed groups at 4-weeks-old (*p* = 1.00). The food intake in the OLETF DT group was significantly lower at 4- and 5-weeks-old during the exercise period than at 6-weeks-old immediately after the exercise period (*p* < 0.001). Body weight was significantly lower in the OLETF DT group than in the OLETF Sed group at 6-weeks-old (*p* < 0.05). The increase in body weight tended to be lower in the OLETF DT group compared to the OLETF Sed group during the exercise period. There were no significant differences in body weight between the OLETF DT and LETO Sed groups from 4- to 5-weeks-old (*p* = 1.00). Body weight was significantly lower in the OLETF DT group than in the OLETF Sed group at 6-weeks-old (*p* < 0.05). The food intake in the OLETF DT group increased after the exercise period (i.e., during the non-exercise detraining period), and there were no significant differences in body weight between the OLETF DT and OLETF Sed groups after 7-weeks-old (*p* = 1.00). At 12-weeks-old, the mean body weights were almost the same in both the OLETF DT and OLETF Sed groups.

There were no significant differences in daily running time (*p* = 0.53, Figure 1C) and running distance (*p* = 0.07, Figure 1D) between the OLETF Ex (i.e., exercise from 10- to 12-weeks-old) and OLETF DT (i.e., exercise from 4- to 6-weeks-old) groups.

### 2.2. Skeletal Muscle Responses to Voluntary Wheel Running

**Figure 2:**
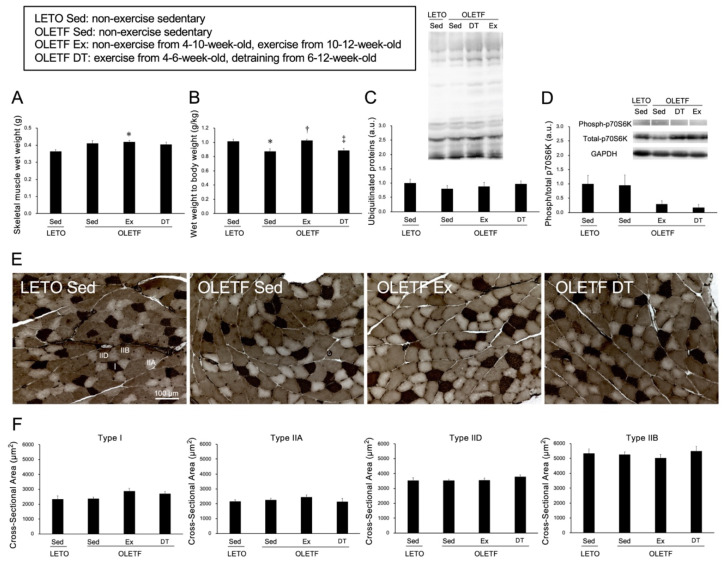
Histological influences of exercise on the skeletal muscle. (**A**) wet weight of the skeletal muscle; (**B**) the ratio of muscle wet weight to body weight; (**C**) expression level of ubiquitinated proteins; (**D**) phosphorylation of p70S6K; (**E**) representative sections of skeletal muscle stained with adenosine triphosphatase (ATPase) (pH 4.6); (**F**) cross-sectional area of type I, IIA, IID, and type IIB fibers. I: Type I fiber; IIA: Type IIA fiber; IID: Type IID fiber; IIB: Type IIB fiber. Values represent means ± standard deviation. For the expression level of protein (**C**,**D**) values are calculated as the fold changes relative to the LETO Sed group. *, †, and ‡ show significantly different from the LETO Sed, OLETF Sed, and OLETF Ex groups, respectively, *p* < 0.05.

There were no significant differences in skeletal muscle wet weight between the OLETF Sed and LETO Sed groups (*p* = 0.11, Figure 2A). The ratio of muscle wet weight to body weight was significantly lower in the OLETF Sed group than in the LETO Sed group (*p* < 0.05, Figure 2B). However, there were no significant differences in the expression levels of ubiquitinated proteins between the OLETF Sed and LETO Sed groups (*p* = 1.00, Figure 2C).

Skeletal muscle wet weight was significantly higher in the OLETF Ex group than in the LETO Sed group (*p* < 0.05). However, there were no significant differences in the ratio of muscle wet weight to body weight between the OLETF Ex and LETO Sed groups (*p* = 1.00). Additionally, there were no significant differences in p70S6K phosphorylation between the OLETF Ex and LETO Sed groups (*p* = 0.32, Figure 2D).

Adenosine triphosphatase (ATPase) staining revealed that the plantaris muscles were composed of type I, IIA, IID, and IIB fibers (Figure 2E), and no obvious differences in muscle fiber type distribution were observed among all groups. Additionally, obvious ectopic lipid accumulation in the skeletal muscle was not observed in the OLETF rats. In all muscle fiber types, there were no significant differences in the cross-sectional area among the groups (*p* = 1.00, Figure 2F).

### 2.3. Exercise and Detraining Effects on Brown Adipose Tissue (Including Chronic Training)

**Figure 3:**
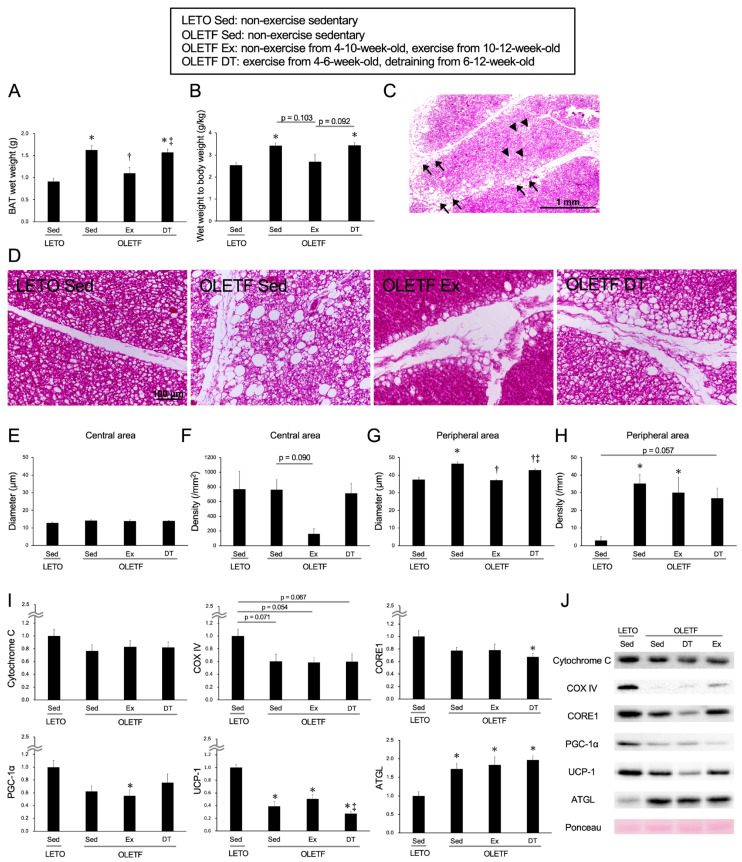
Chronic influences of exercise on brown adipose tissue at 12-weeks-old. (**A**) Wet weight of the brown adipose tissue; (**B**) Ratio of brown adipose tissue wet weight to body weight; (**C**) Representative section of brown adipose tissue stained with hematoxylin and eosin in the OLETF Sed group; (**D**) Representative sections in the peripheral area of the adipose lobule; (**E**) Diameter of whitened unilocular adipocyte in the central area of the lobule; (**F**) Density of whitened unilocular adipocyte in the central area of the lobule; (**G**) Diameter of hypertrophic unilocular adipocyte in the peripheral area of the lobule; (**H**) Density of hypertrophic unilocular adipocyte in the peripheral area of the lobule; (**I**) Expression levels of the proteins in the brown adipose tissue at 12-weeks-old; (**J**) Representative images for the proteins. OLETF DT, OLETF exercise from 4- to 6-weeks-old and non-exercise detraining from 6- to 12-weeks-old. Arrowheads indicate whitened unilocular adipocytes located in the central area of the adipose lobule. Arrows indicate hypertrophic unilocular adipocytes located in the peripheral area of the adipose lobule. Values represent means ± standard deviation. *, †, and ‡ show significantly different from the LETO Sed, OLETF Sed, and OLETF Ex groups, respectively, *p* < 0.05.

The results for the BAT at 12-weeks-old are shown in Figure 3. At 12-weeks-old, the OLETF DT group underwent detraining for 6 weeks after the exercise period.

In terms of both the BAT wet weight (Figure 3A) and the ratio to body weight (Figure 3B), the values were significantly higher in the OLETF Sed group than in the LETO Sed group (*p* < 0.05). In the OLETF Sed group at 12-weeks-old, whitened unilocular adipocytes were observed throughout the adipose lobule (Figure 3D). Sometimes at 12-weeks-old, hypertrophic unilocular adipocytes were observed not only in the peripheral area but also in the central area of the lobule. In terms of both diameter and density of whitened unilocular adipocytes located in the central area, there were no significant differences between the OLETF Sed and LETO Sed groups (diameter: *p* = 0.57, density: *p* = 1.00; Figure 3E,F). In contrast to the central area of the adipose lobule, the diameter and density of hypertrophic unilocular adipocytes located in the peripheral area were higher in the OLETF Sed group than in the LETO Sed group (*p* < 0.01; Figure 3G,H).

The mean values of the BAT wet weight and the ratio to body weight in the OLETF Ex group tended to be lower compared to those in the OLETF Sed group (wet weight: *p* < 0.01), and no significant differences were observed between the OLETF Ex and LETO Sed groups (*p* = 1.00). Although there were no significant differences in the diameter of whitened unilocular adipocytes located in the central area between the OLETF Ex and OLETF Sed groups (*p* = 1.00), the density appeared to be lower in the OLETF Ex group than in the OLETF Sed group. Additionally, in terms of the diameter of hypertrophic unilocular adipocytes located in the peripheral area, the value was significantly lower in the OLETF Ex group than in the OLETF Sed group (*p* < 0.001). However, there were no significant differences in the density of hypertrophic unilocular adipocytes located in the peripheral area between the OLETF Ex and OLETF Sed groups (*p* = 1.00), and the values in OLETF rats tended to be higher compared to those in LETO rats.

Regarding the influence of detraining for 6 weeks, there were no significant differences in both the BAT wet weight and the ratio to body weight between the OLETF DT and OLETF Sed groups (*p* = 1.00). Although the diameter of hypertrophic unilocular adipocytes located in the peripheral area was significantly lower in the OLETF DT group than in the OLETF Sed group (*p* < 0.01), there were no significant differences in the density between the OLETF DT and OLETF Sed groups (*p* = 1.00).

The expression levels of Cytochrome C, COX IV, CORE1, PGC-1α, and UCP-1 were lower in the OLETF Sed group than in the LETO Sed group (Cytochrome C: *p* = 0.528, CORE1: *p* = 0.24, PGC-1α: *p* = 0.092), and significant differences were observed in the expression levels of UCP-1 (*p* < 0.001) (Figure 3I,J). Although the exercise for 2 weeks histologically inhibited BAT whitening in the OLETF rats, there were no significant differences in the expression levels of Cytochrome C, COX IV, CORE1, PGC-1α, and UCP-1 between the OLETF Ex and OLETF Sed groups (Cytochrome C: *p* = 1.00, COX IV: *p* = 1.00, CORE1: *p* = 1.00, PGC-1α: *p* = 1.00, UCP-1: *p* = 0.96). Additionally, there were no significant differences in the expression levels between the OLETF DT and OLETF Sed groups (Cytochrome C: *p* = 1.00, COX IV: *p* = 1.00, CORE1: *p* = 1.00, PGC-1α: *p* = 1.00, UCP-1: *p* = 0.91). Although the expression level of ATGL was significantly higher in the OLETF Sed group than in the LETO Sed group (*p* < 0.01), there were no significant differences in the expression levels among the OLETF Sed, OLETF Ex, and OLETF DT groups (*p* = 1.00).

## 3. Discussion

The results of the present study revealed that childhood exercise inhibits BAT whitening in obesity. Although the inhibition of BAT whitening was observed through detraining, the effect was attenuated during the non-exercise period. The downregulation of UCP-1 was not improved, even though exercise histologically inhibited BAT whitening in obesity.

The present study showed that exercise from 10- to 12-weeks-old decreased whitened adipocyte density located in the central area and the diameter located in the peripheral area of the adipose lobule. Welly et al. reported that BAT whitening in obesity induced by a high-fat diet is inhibited by low-intensity aerobic exercise using wheel running every day for 14 weeks, from 4- to 18-weeks-old [14]. Skeletal muscle hypertrophy was not found in the present study, suggesting that voluntary wheel running was also a low-intensity aerobic exercise. Therefore, in addition to lower food intake during the exercise period, exercise could activate fatty acid oxidation on whitened unilocular adipocytes in BAT, and energy dissipation with shrinkage of lipid droplets results in the reduction of whitened adipocyte density and diameter. Additionally, body weight was significantly lower in OLETF rats exercised from 4- to 12-weeks-old than in OLETF rats exercised from 10- to 12-weeks-old, indicating that the changes in the OLETF Ex group are caused by exercise for 2 weeks as an acute effect, and exercise from 10- to 12-weeks-old could be enough to decrease BAT whitening.

At 12-weeks-old, the diameter of hypertrophic whitened adipocytes located in the peripheral area of the adipose lobule was significantly lower in the OLETF DT group than in the OLETF Sed group. However, no significant differences were observed in body weight and BAT wet weight between the OLETF DT and OLETF Sed groups. These results suggest that short-term exercise in immature rats maintains the size of adipocytes at a smaller level even during the non-exercise detraining period (i.e., slight carry-over effect). Although we found BAT whitening characterized by hypertrophic unilocular adipocytes in the OLETF Sed group at 6-weeks-old, the whitening was completely inhibited in the OLETF DT group at 6-weeks-old immediately after the exercise period (data not shown). In the OLETF DT group, excessive weight gain with hyperphagia was observed after the exercise period (not weight regaining), and BAT whitening was observed at 12-weeks-old after 6 weeks of detraining. The obesity durations in the OLETF Sed and OLETF DT groups were 5- to 12-weeks-old and 7- to 12-weeks-old, respectively; the periods were almost the same. Kwon et al. reported that individuals who decline regular physical exercise in childhood have a higher rate of obesity in adulthood than individuals who maintain physical activity and exercise throughout childhood and adolescence [32]. The decline in physical activity was often found by the age of 6–7 years in the case of obesity [33]. Therefore, even though there is a difference between immature rats and human children, regular physical exercise should be initiated early and continued throughout childhood.

BAT whitening was histologically observed and downregulation of UCP-1 occurred in non-exercise sedentary OLETF rats at 12-weeks-old. Although exercise from 10- to 12-weeks-old histologically inhibited BAT whitening in OLETF rats, downregulation of UCP-1 was observed regardless of exercise and sedentary activity. Previous studies have reported not only hypertrophic whitened adipocytes but also UCP-1 depletion in 20-week-old obese mice induced by a high-fat diet for 14 weeks [8,28]. Downregulation of mitochondrial proteins was found in white adipose tissue with excessive hypertrophic lipid droplets due to the production of reactive oxygen species [34]. Additionally, Shimizu et al. reported that downregulation of mitochondrial proteins in BAT coincided with large lipid droplet accumulation and elevated mitochondrial reactive oxygen species production [10]. The diameter of hypertrophic whitened adipocytes located in the peripheral area of the adipose lobule was significantly lower in the OLETF Ex group than in the OLETF Sed group, the values in the OLETF Ex and LETO Sed groups were almost the same. However, there were no significant differences in the density between the OLETF Ex and OLETF Sed groups, and the value was significantly higher in the OLETF Ex groups than in the LETO Sed. Therefore, in addition to abnormal size, the number of hypertrophic whitened adipocytes may be associated with the downregulation of UCP-1.

Although exercise from 10- to 12-weeks-old improved BAT whitening, the exercise period could be insufficient to induce mitochondrial biogenesis. A previous study reported downregulation of PGC-1α and UCP-1 on BAT in OLETF rats at 10-weeks-old [35], suggesting that downregulation of mitochondrial proteins on brown adipocytes had already occurred in the OLETF Ex group before the beginning of exercise period. Upregulation of PGC-1α and UCP-1 in BAT induced by aerobic exercise for 8 weeks was observed in non-obese individuals [36], and also in obesity [37]. Therefore, long-term regular exercise may be needed to induce mitochondrial biogenesis in obese patients with BAT whitening.

The present study demonstrated the effectiveness of exercise on BAT whitening in obesity. Nevertheless, this study has certain limitations. The mechanism by which BAT whitening causes downregulation of mitochondrial proteins has not yet been elucidated. Further research is required to ascertain the time course of BAT function during exercise and detraining. A study to determine the optimum exercise period is also needed to improve downregulation of mitochondrial proteins in BAT whitening. In the present study, daily running time and running distance were lower in the OLETF Ex group than in the OLETF DT group. OLETF rats as mature adults hardly ever run if they do not have a familiarization period [38]. No familiarization period in immature stage could result in less running time and distance. Finally, this study is based on a small sample size. The power calculation indicated that a maximum sample size of *n* = 56 was required to detect significant differences (not a strong tendency) among the experimental groups in Figure 3B,F,H,I. Further research with a large sample size is needed to reinforce our findings.

## 4. Materials and Methods

### 4.1. Experimental Design

**Figure 4:**
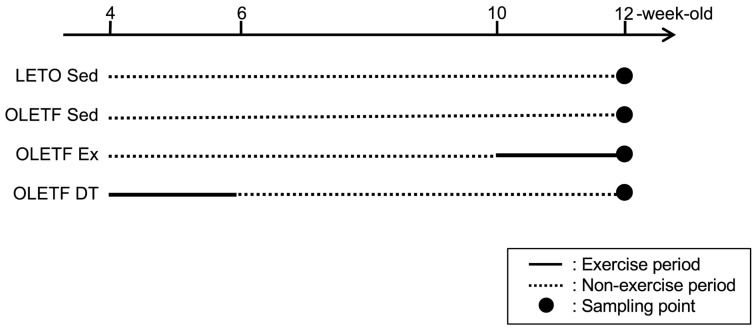
Experimental design. LETO Sed, LETO non-exercise sedentary; OLETF Sed, OLETF non-exercise sedentary; OLETF Ex, OLETF non-exercise from 4- to 10-weeks-old and exercise from 10- to 12-weeks-old; OLETF DT, OLETF exercise from 4- to 6-weeks-old and detraining from 6- to 12-weeks-old. Dark circles indicate the point of tissue sampling. Solid and dotted lines indicate exercise and non-exercise periods, respectively. Tissue samples were collected at 6- (*n* = 3 in the LETO Sed, OLETF Sed, and OLETF DT groups) or 12 (*n* = 6 in each group)-weeks-old. Tissue samples at 6-weeks-old were used to confirm the development process of BAT whitening (data not shown).

Four-weeks-old male Long-Evans Tokushima Otsuka (LETO) rats (78 ± 2 g, *n* = 9) and age-matched male Otsuka Long-Evans Tokushima Fatty (OLETF) rats were used as non-obese and obese animals, respectively. OLETF rats have a spontaneous mutation of the cholecystokinin-1 receptor in the ventromedial nucleus of the hypothalamus, which results in hyperphagia and obesity [39]. OLETF rats were randomly assigned to either the non-exercise sedentary (OLETF Sed, 86 ± 2 g, *n* = 9) or exercise groups. OLETF rats in the exercise group were further divided into subgroups according to the exercise period: non-exercise from 4- to 10-weeks-old, exercise from 10- to 12-weeks-old (OLETF Ex, 96 ± 2 g, *n* = 6), and exercise from 4- to 6-weeks-old, and detraining from 6- to 12-weeks-old (OLETF DT, 93 ± 2 g, *n* = 9). Three rats were placed in a cage and housed in a controlled room with 12-h light and dark cycle at a constant temperature of 22 ± 2 °C. Food and water were provided ad libitum. The rats were sacrificed with an overdose of sodium pentobarbital, and tissue samples were removed at 6- (*n* = 3 in the LETO Sed, OLETF Sed, and OLETF DT groups) and 12-weeks-old (*n* = 6 in each group). Tissue samples at 6-weeks-old were used to confirm the development process of BAT whitening (data not shown). Tissue samples at 12-weeks-old were used to clarify influences of exercise and detraining (Figure 4).

Additionally, influence of long-term exercise period was investigated as a supplementary experiment. Six male OLETF rats performed the exercise from 4- to 12-weeks-old (OLETF Ex4–12w, 102 ± 1 g, *n* = 6). The rats in the OLETF Ex4–12w group was used to detect the difference between exercise from 10- to 12-weeks-old and 4- to 12-weeks-old.

This study was approved by the Institutional Animal Care and Use Committee of Hiroshima University (A19-163) and was performed according to the Hiroshima University Regulations for Animal Experimentation. All experiments were conducted according to the National Institute of Health Guidelines for the Care and Use of Laboratory Animals.

### 4.2. Voluntary Wheel Running Exercise Protocol

OLETF rats in the exercise group were singly housed in cages with a freely accessible running wheel for 12 h (20:00 to 8:00) daily. The rats could run anytime by using the running wheel. Voluntary wheel running was recorded as the running time and distance for 12 h. Food and water were provided ad libitum in cages with running wheels.

### 4.3. Tissue Sampling

The plantaris muscle from the calf and BAT from the interscapular region were collected and weighed at 12-weeks-old. The samples of skeletal muscle and right BAT were removed immediately from each rat, frozen in liquid nitrogen, and stored at −80 °C until further analysis. The left BAT was subsequently removed, fixed with 4% paraformaldehyde in 0.1 M phosphate buffer, and embedded in paraffin. At 12-weeks-old, samples were collected at 5 days after exercise.

### 4.4. Analysis of the Skeletal Muscle

Serial transverse sections in the middle part of the muscle belly were obtained using a cryostat. The sections were stained for myofibrillar ATPase (pH 4.6) and succinate dehydrogenase (SDH) activities to categorize the muscle fibers as type I, IIA, IID, or IIB, based on our previous study [40]. The sections stained with ATPase and SDH were used to determine the composition of muscle fiber types and to measure the cross-sectional area of the muscle fibers of each type. For the measurement of the cross-sectional area of muscle fibers, two random fields were chosen in the superficial and deep layers of the muscle, and >150 muscle fibers were measured per rat. The muscle fiber cross-sectional areas of each type were quantified using ImageJ software (NIH, Rockville, MD, USA).

The skeletal muscle samples were homogenized in extraction buffer (PRO-PREP, iNtRON Biotechnology, Burlington, MA, USA) and 1% phosphatase inhibitor cocktail (07575-51, Nacalai Tesque, Kyoto, Japan). The homogenates were centrifuged at 13,000 rpm for 10 min at 4 °C. Total protein was collected from the supernatant, and the concentration was determined using a protein determination kit (500-0006, Bio-Rad, Hercules, CA, USA). The homogenates were solubilized in a sample loading buffer (196-11022, Wako, Osaka, Japan) and boiled for 10 min at 80 °C. Forty or 60 micrograms of sample protein was loaded on SDS-PAGE, and the proteins were transferred to a PVDF membrane. After blocking in a blocking reagent (Blocking One-P, Nacalai Tesque, Kyoto, Japan) for 60 min at room temperature, the membranes were incubated with anti-total p70S6K (1:1000; 2708, Cell Signaling, Burlington, MA, USA), anti-phospho p70S6K (Thr^389^) (1:1000; 9205, Cell Signaling), anti-ubiquitinated proteins (1:500; A-106, Boston Biochem, Cambridge, MA, USA), and anti-Glyceraldehyde-3-phosphate dehydrogenase (GAPDH) (1:200; sc-32233, Santa Cruz Biotechnology, Dallas, TX, USA) antibodies for 12 h at 4 °C. Subsequently, the membranes were incubated for 60 min at room temperature with anti-rabbit (1:10,000; 111-035-003, Jackson ImmunoResearch, West Grove, PA, USA) or anti-mouse IgG (1:10,000; 1030-05, SouthernBiotech, Birmingham, AL, USA) antibody conjugated to horseradish peroxidase. The signals were detected using a chemiluminescence detector (ECL Select, Merck, Darmstadt, Germany) and analyzed using an image reader (Versa Doc 5000, Bio-Rad, Hercules, CA, USA).

### 4.5. Analysis of the Brown Adipose Tissue

Transverse sections were obtained from the middle part of the BAT using a microtome. The sections were stained with hematoxylin and eosin for histological observation. Additionally, the images were used to measure the diameter and density of whitened brown adipocytes in BAT. For the measurements, two random fields were chosen in the center and peripheral areas of the adipose lobule. Unilocular adipocytes with a diameter >10 µm were defined as whitened adipocytes in the center areas of the adipose lobule. Enlarged unilocular adipocytes with a diameter >30 µm were defined as hypertrophic whitened adipocytes in the peripheral areas of the adipose lobule. The adipocyte density in the center area was calculated as the number per unit area (number/mm^2^). In the peripheral area, the density was calculated as the number of adipocytes located in the perimeter of the lobule (number/mm). The adipocyte diameter and adipocyte density were quantified using ImageJ software (NIH, Rockville, MD, USA).

The samples in the middle part of the BAT were homogenized in extraction buffer containing 50 mM Tris-HCl (pH 7.8), 0.15 M NaCl, and 1% protease inhibitor cocktail (25955-11, Nacalai Tesque, Kyoto, Japan). The homogenates were centrifuged at 5200 rpm for 5 min at 4 °C. The homogenates collected from the supernatant were solubilized in a sample loading buffer (196-11022, Wako, Osaka, Japan) and boiled for 10 min at 80 °C. Ten micrograms of sample protein was loaded on SDS-PAGE, and the proteins were transferred to a PVDF membrane. Following blocking step in a blocking reagent (Blocking One, Nacalai Tesque, Kyoto, Japan) for 60 min at room temperature, incubation with anti-Cytochrome C (1:1000; 33-8500, Thermo Fisher Scientific, Waltham, MA, USA), anti-Cytochrome C oxidase subunit IV (COX IV) (1:1000; 4850, Cell Signaling), anti-Ubiquinol-Cytochrome C reductase core protein I (CORE1) (1:2000; ab110252, Abcam, Cambridge, MA, USA), anti-PPAR*γ* co-activator-1α (PGC-1α) (1:200; sc-13067, Santa Cruz Biotechnology), anti-UCP-1 (1:1000; ab10983, abcam), and anti-Adipose triglyceride lipase (ATGL) (1:1000; 2138, Cell Signaling) primary antibodies for 12 h at 4 °C, and incubation with anti-rabbit (1:10,000; 111-035-003, Jackson ImmunoResearch) or anti-mouse IgG (1:10,000; 1030-05, SouthernBiotech) secondary antibody conjugated to horseradish peroxidase for 60 min at room temperature, the signals were detected using a chemiluminescence detector (ECL Prime, Merck) and analyzed with an image reader (Versa Doc 5000, Bio-Rad).

### 4.6. Statistical Analysis

The data are expressed as the mean ± standard deviation. The significance of differences between the groups was evaluated using a one-way analysis of variance, followed by Bonferroni’s honest significant difference post hoc test. For the results of body weight and food intake, the main effect of the experimental group, weeks of age, and the interaction were evaluated using two-way analysis of variance, followed by Bonferroni’s honest significance difference post hoc test. Statistical significance was set at *p* < 0.05. All statistical analyzes were performed using SPSS statistical analysis software (IBM SPSS Statistics version 19.0, IBM Japan, Tokyo, Japan). Statistical power were analyzed by G*Power (Ver. 3.1.9.6, Heinrich Heine University of Dusseldorf, Germany).

## 5. Conclusions

Although childhood exercise inhibits BAT whitening in obesity, regular exercise would have to continue to improve downregulation of mitochondrial proteins in BAT. BAT whitening progresses rapidly during non-exercise detraining in hyperphagia. These results suggest that exercise in childhood should be continued to inhibit BAT whitening and prevent obesity in adulthood.

## Data Availability

The data presented in this study are available in article.
